# Influence of Inter-Pass Cooling on Microstructural Evolution and Plastic Deformation of Heavy EH47 Plates

**DOI:** 10.3390/ma12101686

**Published:** 2019-05-23

**Authors:** Junyu Wu, Bin Wang, Bingxing Wang, R. D. K. Misra, Zhaodong Wang

**Affiliations:** 1 State Key Laboratory of Rolling and Automation, Northeastern University, Shenyang 110819, China; 1410174@stu.neu.edu.cn (J.W.); wangbin404@126.com (B.W.); zhdwang@mail.neu.edu.cn (Z.W.); 2 Laboratory for Excellence in Advanced Steel Research, Department of Metallurgical, Materials and Biomedical Engineering, University of Texas at El Paso, 500 W. University Avenue, El Paso, TX 79968, USA; dmisra2@utep.edu

**Keywords:** inter-pass cooling (IC), heavy EH47 plate, grain refinement, ferrite and bainite, FCC, synergetic plastic deformation

## Abstract

Herein, the influence of inter-pass cooling (IC) and conventional two-stage rolling (CTR), on microstructural evolution and plastic deformation behavior of ultra-heavy EH47 plates, is demonstrated. It is reported that the deformation amount and deformation rate, in every deformation pass during rough rolling, at 1/4- and 1/2-thickness of IC steel were higher than the CTR steel. The volume fraction of ferrite and acicular ferrite was 45% and 18%, at 1/4-thickness, and 35% and 50% at 1/2-thickness of IC steel, respectively, whereas the sum of both ferrite phases was smaller than 25% in the CTR steel. The austenite grain boundary area and high-angle grain boundary fraction in the IC steel were higher than the CTR steel. The high density of fine and shapeless pearlite has been observed in IC steel, whereas large-size carbides, with hexagonal structure, have been observed in CTR steel. Compared to the CTR steel, the density of precipitates was apparently lower in IC steel. Two kinds of Nb containing precipitates, such as (Ti, Nb)(C, N) and (Nb, Ti)C, were observed in the tested steels. Total ductility and uniform elongation of the IC steel were higher than the CTR steel. During the tensile process, the crack initiation energy and crack propagation energy of the IC steel were higher than the CTR steel. Moreover, the volume fraction of retained austenite (FCC) was reduced from 7.71% to 0.42% near the tensile fracture in IC steel at 1/4-thickness. In additon, the strain of synergetic plastic deformation of the IC steel was higher than the CTR steel. Meanwhile, compared to the CTR steel, the synergetic plastic deformation of the IC steel occurred at low stress after the yield point, which can be ascribed to the presence of fewer microcracks in the IC steel. Hence, a delayed fracture has been observed in the IC steel plate.

## 1. Introduction

Shipping containers play an important role in international trade [1]. To increase the freight capacity and reduce energy consumption, container ships need to be large, which requires thicker and stronger upper steel deck for safety [2,3]. However, with the increasing thickness and strength of the heavy steel plate, the crack resistance ability is decreased [4] and ductile-brittle transition temperature (DBTT) is increased [5,6]. Therefore, in order to avoid the damage of the container ship, heavy steel plates are required, with high-strength and high crack resistance [7].

To satisfy the strength requirements, high content of bainite, with a high density of dislocations, M-A islands and carbides, is introduced in traditional high-strength ship-building steel plates. During the plastic deformation of steel plate, the stress concentration originates from dislocation pile-up and uncoordinated deformation [8,9,10] between the matrix and the hard phase (M-A islands and carbides), which leads to the brittle fracture. In contrast, as a product of diffusive transformation, ferrite grains contain fewer dislocations because of the high phase transformation temperature and smaller grain size due to the high nucleation rate. Therefore, in the deformed plate with ferrite matrix, stress concentration, caused by dislocation pile-up is difficult to occur and crack propagation energy is higher due to the inhibition and deflection behavior, which is introduced by the higher concentration of high-angle grain boundaries (GBs). Furthermore, the interlaced lath of acicular ferrite has a mis-orientation higher than 15° and can be easily refined with the increase of austenite deformation band density [11]. Thus, the acicular ferrite inhibits and deflects the propagation of brittle cracks [12,13]. Most importantly, the multiphase steel plates, with bainite and ferrite structures, render high plastic deformation and crack resistance compared to the bainite steel plates. It is worth mentioning that the grain refinement of ferrite enhances the strength of the resulting steel, which compensates the loss of strength due to the reduced density of dislocations, M-A islands and carbides.

The deformation penetration of conventional two-stage rolling (CTR) process can hardly satisfy the deformation requirement in the core of the ultra-heavy plate due to the low rolling reduction rate. Hence, CTR results in low strength and toughness. Compared to CTR, the inter-pass cooling (IC) process [4,14,15] is considered advantageous because it can form large temperature gradients along the thickness direction of the slab, before the rolling passes, by using the cooling device beside the rolling mill, which improves the deformation penetration and refines the austenite grains inside the slab [16]. Moreover, the polygonal ferrite and acicular ferrite are formed at the austenite grain boundary and deformation bands during the cooling process, which can be further refined to attain high performance steel plate.

Recently published studies have demonstrated the deformation permeability of heavy plate in the hot rolling process using IC technology [14,15,16]. However, the influence of the IC process, on microstructure evolution and tensile property of heavy plates, has not been reported yet. In the present study, two ultra-heavy EH47 plates were rolled from the thickness of 160 mm to 60 mm and the IC process was introduced during rough rolling of one of the plates. The influence of the IC process on microstructural evolution and plastic deformation inside the plates has been systematically investigated.

## 2. Experimental Procedure

### 2.1. Hot Rolling Experiments

The plates were rolled on a 750 mm experimental hot rolling mill and IC was carried out by using a cooling device beside the rolling mill. The chemical composition of the EH47 steels plate was: 0.05 C, 1.69 Mn, 0.12 Si, 0.015 Ti, 0.044 Nb, 0.15 Cr, 0.2 Cu, 0.48 Ni, 0.03 Al and 0.003 N (wt.%). The 160 mm thick slab was heated to 1200 °C for 4 h to dissolve the micro-alloying elements. The reduction schedule was: 160-140-122-106-94-84-74-65-60 (mm), while the finish rolling starting thickness was 106 mm.

**Figure 1:**
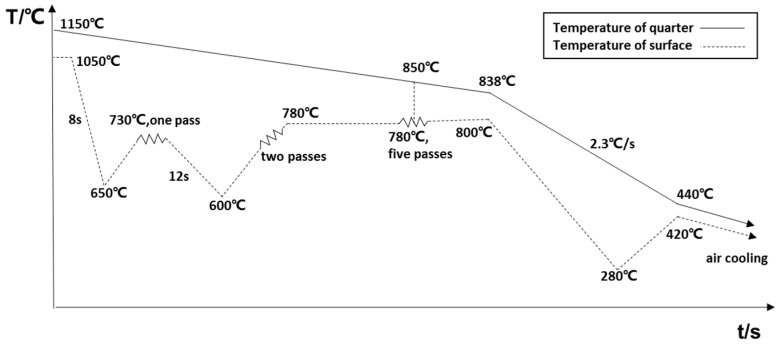
Schematic illustration of the inter-pass cooling (IC) process.

The schematic illustration of the IC process is presented in Figure 1. The solid line presents the temperature of the thermocouple inserted at 1/4-thickness of the slab. The temperature was measured with a midi LOGGERGL40 temperature collector (Wilmington Instrument Co., Wilmington, CA, USA). The dotted line shows the temperature of the slab surface, measured with VF-3000 infrared radiation thermometers. During rough rolling, the initial rolling temperature was ~1050 °C at the surface of the slab, while two cooling passes of 8 s and 12 s were carried out before the first and second rolling passes, respectively, which resulted in adequate temperature gradient along the thickness direction. The intermediate slab was held for 220 s. During finish rolling, the initial rolling temperature at the surface and 1/4-thickness of the slab was ~780 °C and 850 °C, respectively. The finish rolling temperature was ~800 °C on the plate surface. After the finish rolling process, the plate was cooled to 420 °C at a cooling rate of ~2.3 °C/s and, then, air cooled to room temperature. In the two-stage continuous rolling of CTR process, the initial rolling temperature was ~1050 °C and the holding time of the intermediate slab was 460 s. The finish rolling process started at 830 °C and after the completion of finish rolling process, the plate was cooled from 800 °C to 400 °C at a cooling rate of ~2.3 °C/s, followed by air cooling till room temperature.

### 2.2. Microstructural and Mechanical Characterization

Metallographic specimens were cut from the middle of the plates at 1/4- and 1/2-thickness. The surfaces perpendicular to the transverse direction (TD) were polished and etched with 4% Nital solution and supersaturated picric acid solution (at 73 °C) for microscopic observations through optical microscopy (Carl Zeiss Axio Imager Alm, Jena, Germany) and electron probe X-ray microanalysis (JEOL-8530F, Tokyo, Japan). The metallographic specimens were electro-polished by using a solution of 10% perchloric acid and 90% ethanol for observations through electron back-scattered diffraction (EBSD) (Oxford instruments, Abingdon, UK, INCA Crystal). In addition, the thin foils, with a diameter of 3 mm were twin-jet electro-polished by using a solution of 12% perchloric acid and 88% ethanol, at −30 °C, to conduct transmission electron microscope (TEM) (JEM-2100 F, JEOL Ltd., Tokyo, Japan) observation at an accelerating voltage of 200 kV.

The cylindrcal tensile specimens with the size of Φ 12 mm × 120 mm was cut from the middle of the plates at 1/4- and 1/2-thickness and the tensile tests were carried out by using the Instron machine (Instron 5585 H, Norwood, MA, USA), while the crosshead speeds were 3 mm/min, and 15 mm/min, respectively, before and after the yield point. The metallographic specimens were cut from the cross-section of the broken tensile specimens and the surfaces were polished and etched with 4% Nital solution. The microstructure near the tensile fracture was observed by scanning electron microscopy (SEM) (ZEISS ULTRA55, Jena, Germany). 

### 2.3. Three-Dimensional Finite Element Analysis (FEA)

**Figure 2:**
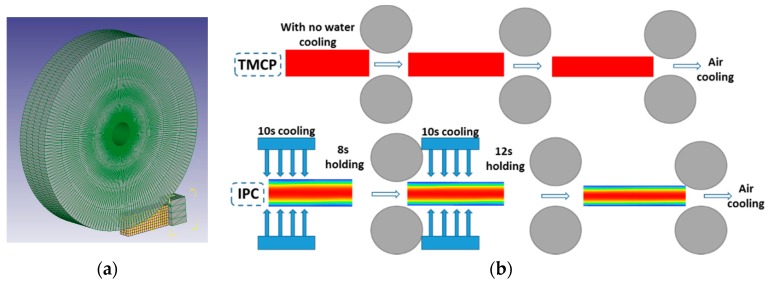
Schematic illustration of (**a**) 1/4 FE geometry model and (**b**) rough rolling of IC and conventional two-stage rolling (CTR) processes. Online version in color.

To study the deformation difference inside the two rolled plates, a three-dimensional (3D) plastic thermo-mechanical FE model, used in the rough rolling process, was developed by using DEFORM-3D program. The roller was defined as a rigid body and the workpiece was considered as a rigid-plastic material with a tetrahedral mesh. The 1/4 FE geometry models are illustrated in Figure 2a and the simulation process is illustrated in Figure 2b.

Based on the Johnson and Cook model and the stress-strain curves, resulting from the single pass deformation experiments using the Gleeble thermo-mechanical simulator, the deformation resistance can be formulated as:

(1) $$\sigma =\left(-9249+9726\varepsilon^{0.0072}\right)\left(1+0.043ln\dot{\varepsilon }\right)\left[1-{\left(\frac{T-20}{1480}\right)}^{1.42}\right]$$

where σ refers to stress (MPa), ε represents the strain, $\dot{\varepsilon }$ corresponds to the strain rate (s^−1^) and *T* denotes the temperature (°C).

The size of roller and workpiece, the rolling reduction and the inter-pass cooling time were same with the experimental values. The shear friction coefficient can be formulated as [17]:

(2) m=1.06−0.0006×T

where m refers to shear friction coefficient, and T denotes the temperature (°C) on the plate surface. The initial conditions of the slab temperature field were homogenous at 1150 °C. By changing the temperature of the plates at 1/4-thickness in accordance with the experimental results, the plate surface heat convection coefficient for air cooling and water cooling processes were set at 0.2 N/s/mm/C, and 5 N/s/mm/C, respectively. The original simulation conditions are shown in Table 1.

## 3. Results and Discussions

### 3.1. Microstructure

**Figure 3:**
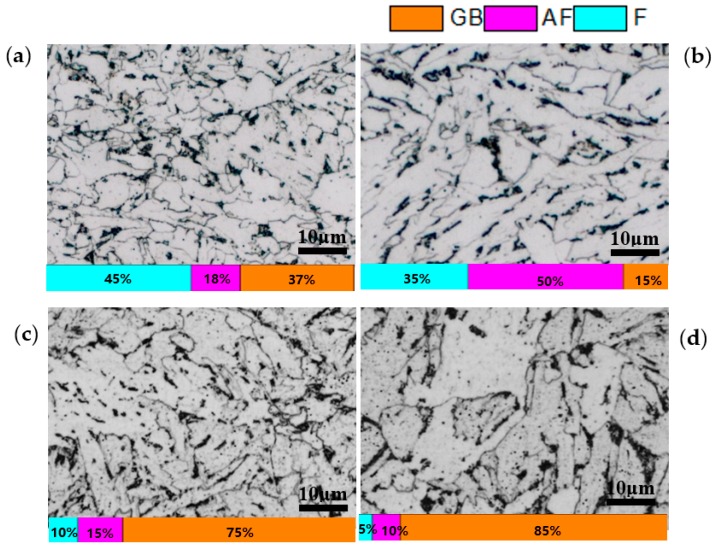
The microstructure at (**a**) 1/4-thickness and (**b**) 1/2-thickness of IC steel; (**c**) 1/4-thickness and (**d**) 1/2-thickness of CTR steel. (GB-granular bainite; AF-acicular ferrite; F-polygonal ferrite and quasi-polygonal ferrite).

**Figure 4:**
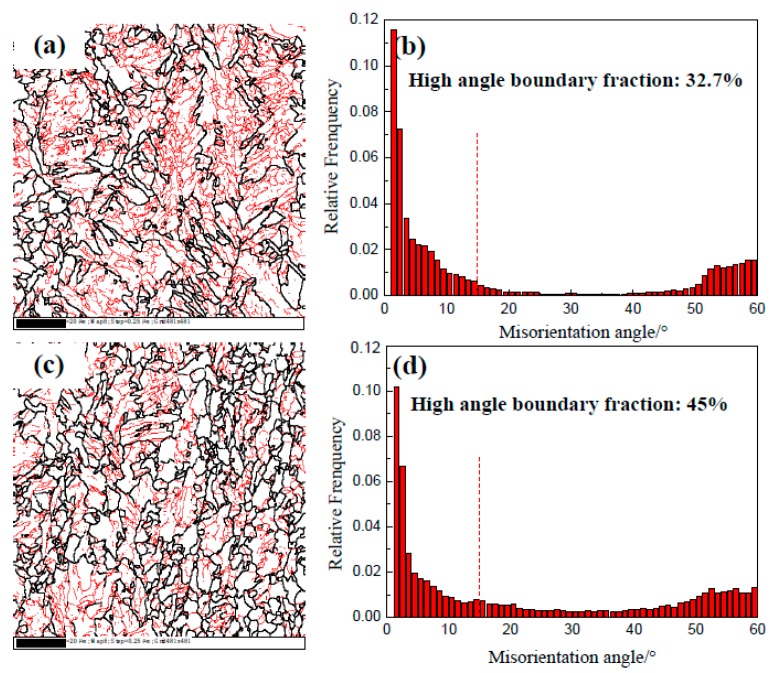
The mis-orientation distribution maps at 1/4-thickness of (**a**) IC and (**c**) CTR plates (black lines represent grain boundaries exceeding 15° and red lines represent grain boundaries between 2° and 15°). The misorientation angle distribution histograms at 1/4-thickness of (**b**) IC and (**d**) CTR plates.

Figure 3 presents the microstructure of IC and CTR steels at 1/4- and 1/2-thickness, while the volume fraction of different microstructural constituents was calculated through multi-view statistics. The figure indicates that the microstructural constituents and grain size of the two plates are significantly different. The microstructure at 1/4- and 1/2-thickness is mainly composed of quasi-polygonal ferrite and acicular ferrite in the IC steel, whereas a high volume fraction of granular bainite has been observed in the CTR steel. From 1/4-thickness to 1/2-thickness of IC steel, the volume fraction of quasi-polygonal ferrite changed from 45% to 35%, whereas the volume fraction of acicular ferrite changed from 18% to 50%. However, the grain size has exhibited a smaller change in the given thickness range. In the CTR steel, the volume fraction of bainite increased from 75% to 85% from 1/4-, to 1/2-thickness, respectively. Also, the grain size increased along the thickness direct and significantly coarsened grains have been observed at 1/2-thickness. Figure 4 presents the mis-orientation maps and mis-orientation angle distributions at 1/4-thickness of the IC and CTR steels, which suggests that both the high angle (>15°) grain boundary area per unit area and high angle area percentage of the total grain boundary (from 2° to 60°) at 1/4-thickness of the IC steel were higher compared to the CTR steel. 

**Figure 5:**
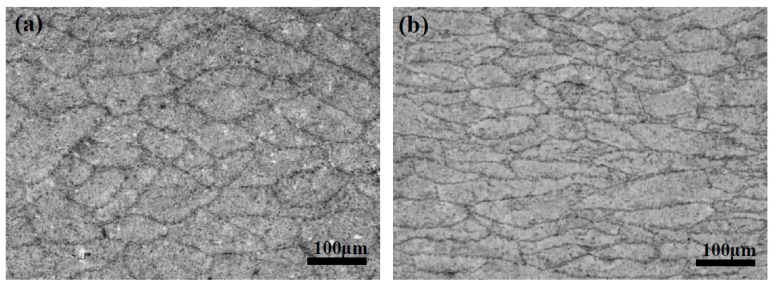
The austenite structure at 1/4-thickness of (**a**) IC and (**b**) CTR plates.

The austenite structure at 1/4-thickness of the two plates is presented in Figure 5, whereas the statistics of the grain size and the number density are shown in Table 2. The results suggest that the number of austenite grains per unit area (mm^2^) was apparently larger in the IC steel (224) than the CTR steel (140). Moreover, the average grain size along the thickness direction was apparently smaller in the IC steel (37 μm) than the CTR steel (70 μm). Also, the average length-width ratio of the austenite grains was higher in the IC steel (3.34) than the CTR steel (2.35).

**Figure 6:**
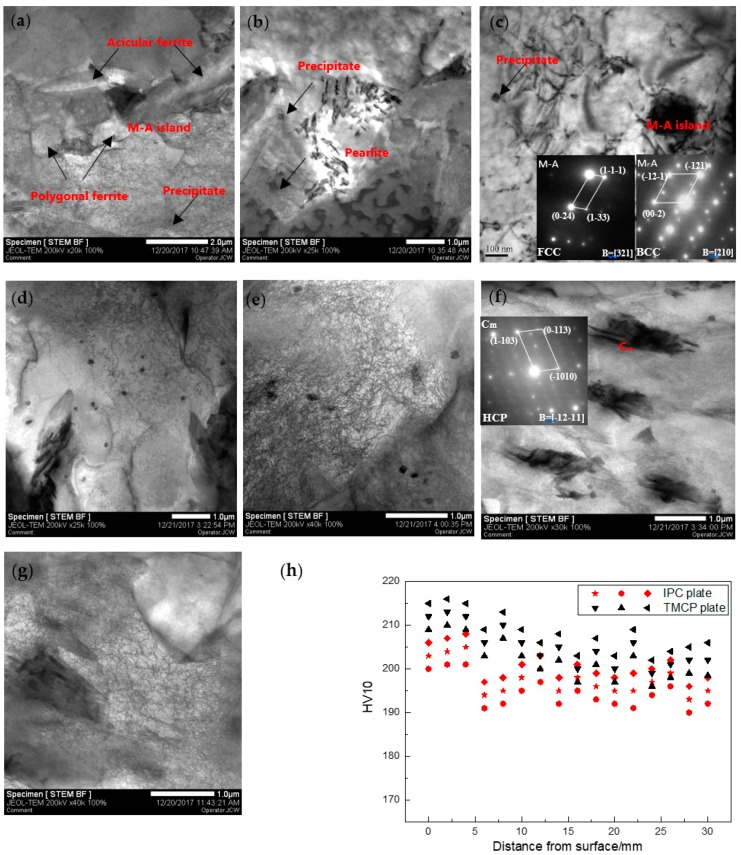
TEM micrographs of precipitates and carbides for (**a**–**c**) IC steel and (**d**–**g**) CTR steel at 1/4-thickness; (**h**) Vickers hardness along the thickness direction of the two plates.

The TEM images of the IC and CTR steels, at 1/4-thickness, are presented in Figure 6a–g. In the IC steel, M-A island was distributed at the grain boundary of the refined acicular ferrite and polygonal ferrite (Figure 6a), the pearlite was fine and shapeless (Figure 6b), and the majority of precipitates were square in shape and the austenite constituent was present in M-A islands (Figure 6c). On the other hand, in CTR steel, pearlite was not been observed, and the large-size carbides of the hexagonal structure were present in bainite lath (Figure 6f). Moreover, several ellipse-shaped precipitates have also been observed in CTR steel. In addition, the grain boundary migration, induced by the expansion force of bainite phase transformation, has also been observed at the interface between bainite and ferrite, resulting in movable dislocations (Figure 6g). Considering that the precipitate number density and carbides were apparently larger in CTR steel compared to IC steel, higher Vickers hardness has been obtained for IC steel than the CTR steel.

**Figure 7:**
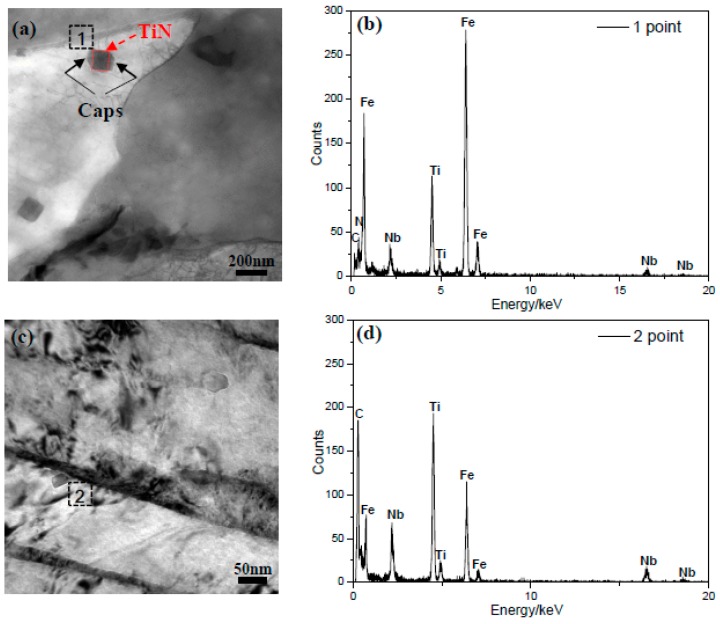
TEM images of the large-sized (**a**) and small-sized (**c**) precipitates; and (**b**,**d**) EDS spectra of the precipitates from two different points.

To further understand the precipitation, the microstructure and EDS (Energy Dispersive Spectrometer) analysis of the precipitates was carried out, and the results are presented in Figure 7. The results reveal that the square-shaped precipitate, surrounded by the caps (Figure 7a), was (Ti, Nb)(C, N), and the small-sized strip-shaped precipitate was (Nb, Ti)C. A number of studies [18,19,20,21] proposed that NbC could nucleate and grow on the TiN surface through diffusion [22] and both NbC and TiN are soluble in each other, due to their identical crystal structure (NaCl type FCC structure) and similar crystal lattice constants (0.447 nm and 0.424 nm) [23]. Hence, the NbC nucleated on TiN precipitate has a spherical shape, which corresponds to the minimal interfacial energy (caps in Figure 7a). Considering the chemical ratio of titanium to nitrogen (3.42) and the composition of the experimental steel, the entire free nitrogen has been consumed and some free titanium (0.006%) has been observed after TiN precipitation, during the soaking process of the slabs. Thus, (Nb, Ti)C precipitate in Figure 7c has been formed when the free titanium was dissolved in pre-precipitated NbC.

### 3.2. Simulation Results

**Figure 8:**
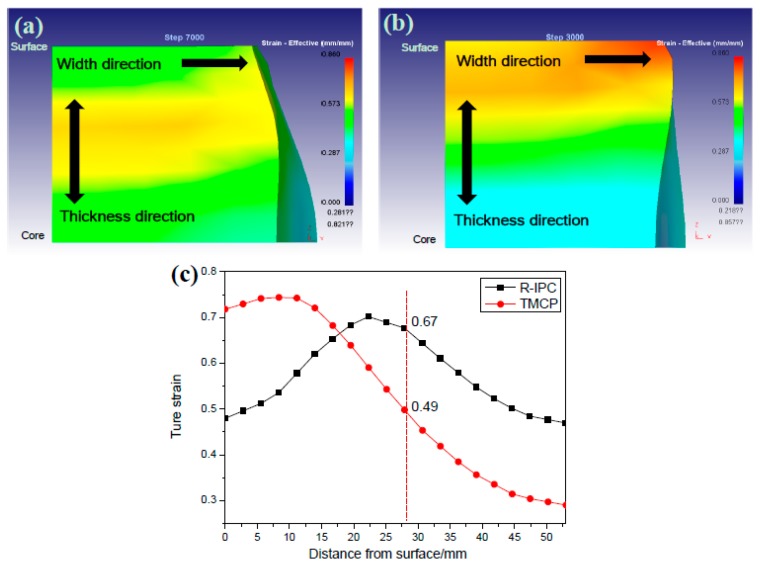
Macroscopic shapes of the end of (**a**) the IC and (**b**) the CTR plates; (**c**) true strain distribution along the thickness direction of the IC and CTR plates.

Figure 8 presents the lateral shapes and through-thickness true strain distribution of the two plates, which suggests that the lateral shapes of the IC and CTR plates exhibit single-drum, and double-drum, respectively. In addition, the cumulative strain was high (>0.7) on 10 mm surface layer, which linearly dropped to a low value (<0.3) from the surface to the core of the CTR plate. On the other hand, the cumulative strain increased from 0.48 (surface) to 0.7 (1/4-thickness) and, then, decreased to 0.47 (1/2-thickness) for the IC plate. Compared to the CTR plate, cumulative strain in the IC plate was higher and the difference increased from 1/6-thickness to 1/2-thickness. Table 3 presents the deformation temperature and deformation rate at 1/4-thickness of the two plates in every deformation pass, which suggests that deformation temperatures were lower and deformation rates were higher for the IC plate than the CTR plate. 

### 3.3. Tensile Results

**Figure 9:**
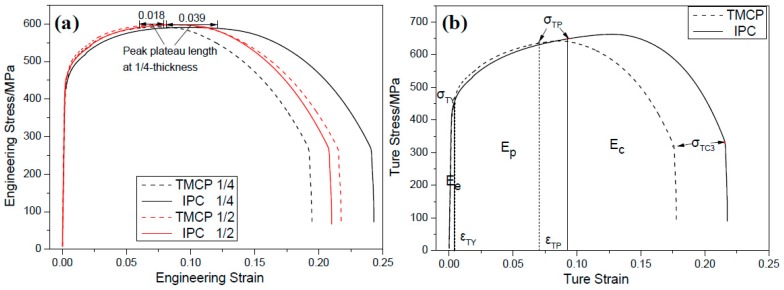
(**a**) Engineering and (**b**) true stress-strain curves at 1/4-thickness of the IC and CTR plates.

Figure 9a presents the engineering stress-strain curves at 1/4- and 1/2-thickness of the two plates, which suggest that ductility and peak plateau length were much larger in the IC steel than in CTR steel at 1/4-thickness. However, this difference was much smaller at 1/2-thickness. 

Figure 9b presents the true stress-strain curves at 1/4- and 1/2-thickness of the two plates, where $\varepsilon_{\mathrm{TY}}$ refers to the yield strain, $\varepsilon_{\mathrm{TP}}$ represents the end strain of uniform plastic deformation, $\sigma_{\mathrm{TY}}$ denotes the yield stress, $\sigma_{\mathrm{TP}}$ corresponds to the stress at the strain of maximum load, $\sigma_{\mathrm{TC}3}$ refers to the triaxial fracture stress, $E_{e}$ represents the elastic deformation energy, $E_{p}$ corresponds to the plastic deformation energy and $E_{c}$ denotes the crack propagation energy. The energy can be expressed through Equations (3)–(5) [24]:

(3) $$E_{e}=\varepsilon_{\mathrm{TY}}\sigma_{\mathrm{TY}}/2$$

(4) $$E_{p}=K\left(\varepsilon_{\mathrm{TP}}{}^{n+1}-\varepsilon_{\mathrm{TY}}{}^{n+1}\right)/\left(n+1\right)$$

(5) $$E_{c}=\left(\varepsilon_{\mathrm{TC}}-\varepsilon_{\mathrm{TP}}\right)\left(\sigma_{\mathrm{TP}}+\sigma_{\mathrm{TC}1}\right)/2$$

where the true fracture strain $\varepsilon_{\mathrm{TC}}$ and true uniaxial fracture stress $\sigma_{\mathrm{TC}1}$ can be expressed by using Equations (6) and (7) [24]:

(6) $$\varepsilon_{\mathrm{TC}}=\ln\left(A_{0}/A_{F}\right)$$

(7) $$\sigma_{\mathrm{TC}1}=\sigma_{\mathrm{TC}3}/\left\{\left(1+1.14/\left(\varepsilon_{\mathrm{TC}}-\varepsilon_{\mathrm{TP}}\right)\right)\ln\left[1+0.88\left(\varepsilon_{\mathrm{TC}}-\varepsilon_{\mathrm{TP}}\right)\right]\right\}$$

where $A_{0}$ refers to original sectional area and $A_{F}$ represents the sectional fracture area. The strain hardening exponent *n* and strength coefficient *K* can be obtained by fitting the Hollomon equation ($\sigma =K\varepsilon^{n}$) of the true stress-strain curves [25]. 

Furthermore, the mechanical parameters and energy distribution at 1/4-thickness of the two plates are presented in Table 4, which suggests that the uniform plastic deformation end strain, crack initiation energy ($E_{e}$ + $E_{p}$) and crack propagation energy ($E_{c}$) of IC steel are larger than the CTR steel. Therefore, compared to the CTR steel, IC steel is harder to form brittle cracks and exhibits superior anti-fracture ability to the plastic deformation process.

**Figure 10:**
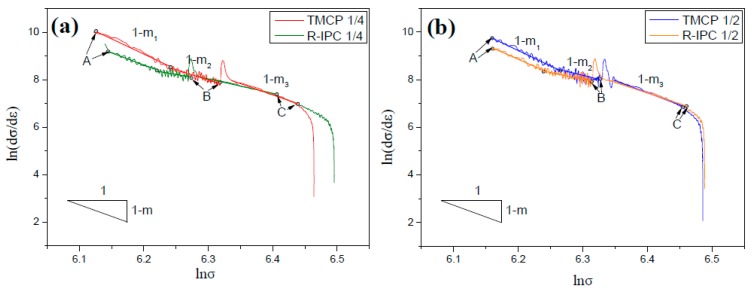
The strain hardening behavior at (**a**) 1/4- and (**b**) 1/2-thickness of the experimental steels, obtained by using the modified C-J analysis.

The C-J analysis was carried out to study the strain-hardening rate during the uniform plastic deformation process. Based on the Swift equation, the derived equation can be expressed by Equation (8) [26,27,28]:

(8) $$\varepsilon =\varepsilon_{0}+C\sigma^{m}$$

where σ refers to the true stress, ε represents the true strain, *m* denotes the stress exponent and C is the material constant. By taking derivative of Equation (8) with respect to σ and, then, expressing it in logarithmic form, the modified C-J analysis can be represented by Equation (9):

(9) ln(dσ/dε)=(1−m)lnσ−ln(Cm)

where strain-hardening rate (dσ/dε) is inversely proportional to stress exponent (*m*). The C-J expression was derived by integrating the current experimental data and the curves, as shown in Figure 10. One should note that “1 − *m*” refers to the slope, point “A” represents the yield starting point, point “B” denotes the synergetic plastic deformation starting point, and point “C” corresponds to the necking starting point.

Previous studies [29,30] have shown that three linear stages exist between ln(dσ/dε) and lnσ in the continuous yield process of ferrite/bainite dual-phase steel: (1) plastic deformation of ferrite and elastic deformation of bainite; (2) restrained plastic deformation of ferrite and elastic deformation of bainite, and (3) synergistic plastic deformation of ferrite and bainite. One should note that the current experimental results (Figure 10) are consistent with the previous studies. Moreover, the slope of three linear stages are named as *m*_1_, *m*_2,_ and *m*_3_, respectively. The critical parameters, derived from Figure 10, are summarized in Table 5, which indicates that *m*_1_ > *m*_3_ > *m*_2_ and the value of *m*_1_–*m*_3_ is larger for IC steel than the CTR steel at 1/4- and 1/2-thickness. In other words, the strain-hardening rate was the highest during the second stage of the continuous yield process, whereas it was lowest in the first stage. Moreover, the strain-hardening rate of IC steel was higher than the CTR steel and the strain of synergistic plastic deformation for IC steel was larger than the CTR steel at 1/4-thickness. However, the difference between yield stress and synergistic plastic deformation starting stress for CTR steel was larger than the IC steel, which suggests that synergistic plastic deformation of IC steel can occur at a lower stress after yielding point. Therefore, less micro-cracks, derived from the uncoordinated deformation, were created in the IC steel, which delays the fracture of IC steel.

**Figure 11:**
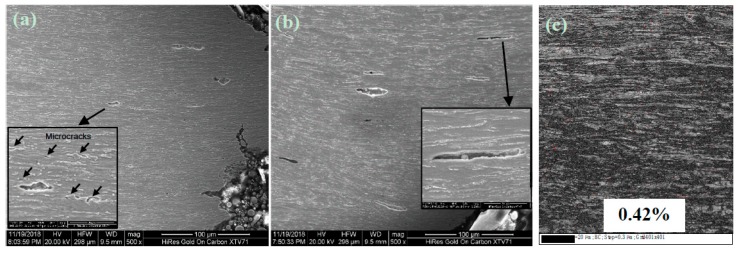
The cross-sectional SEM images near the tensile fracture at 1/4-thickness of (**a**) IC steel and (**b**) CTR steel; (**c**) FCC phase (the red painted part) distribution near the tensile fracture at 1/4-thickness of the IC steel.

In order to further understand the necking process of the two steel plates, the cross-sectional SEM morphology and FCC phase distribution near the tensile fracture, at 1/4-thickness of both plates, are presented in Figure 11. The number density and size of the cracks in the CTR steel were larger than the IC steel at 1/4-thickness, which explains the superior ductility of IC steel. Meanwhile, a large number of micro-cracks have been observed near the tensile fracture at 1/4-thickness of the IC steel. Besides, FCC (face center cubic) volume fraction was only 0.42% near the tensile fracture at 1/4-thickness of the IC steel. 

## 4. Discussion

### 4.1. Phase Transformation

**Figure 12:**
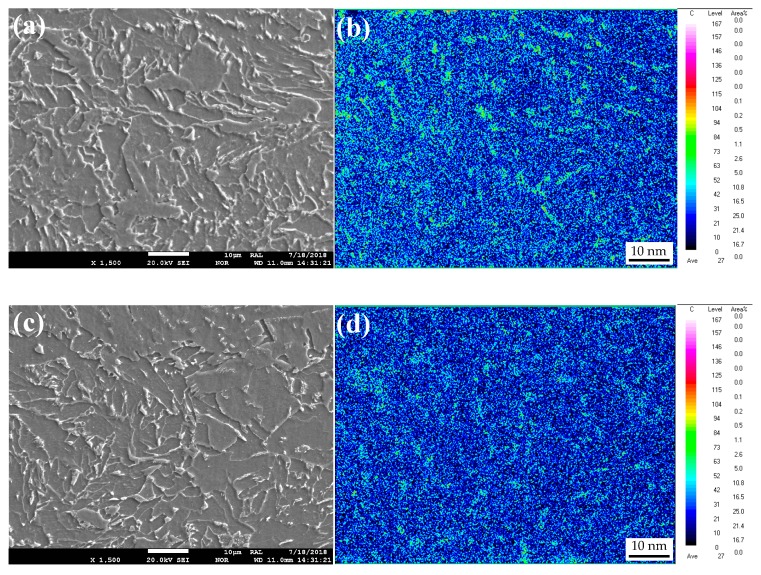
SEM micrographs at 1/4-thickness of (**a**) IC and (**c**) CTR steel plates; and the carbon distribution, measured by using an electronic probe, at 1/4-thickness of (**b**) IC and (**d**) CTR steel plates.

**Figure 13:**
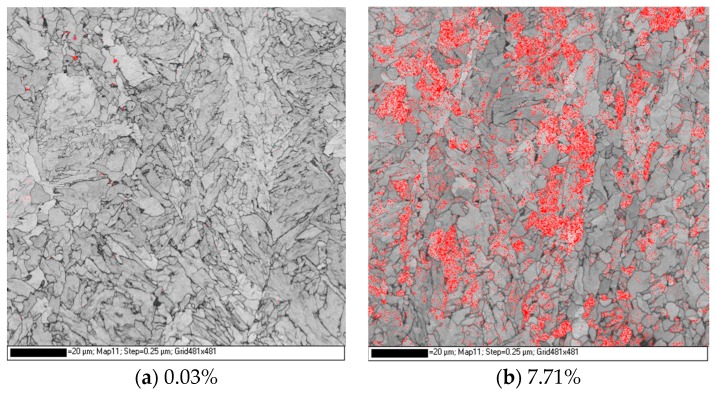
Fraction of retained austenite (FCC) phase (the red painted part) distribution at 1/4-thickness of the (**a**) CTR and (**b**) IC plates.

Figure 12 presents the SEM images and carbon distribution at 1/4-thickness of the two steel plates, which suggested that the carbon intensity of M-A islands and pearlite at the grain boundary and carbon concentration area were greater in the IC steel than the CTR steel. To study the phase composition of the carbon concentration regions, EBSD analysis was carried out and the FCC phase distribution is shown in Figure 13, which suggested that the volume fraction of the retained austenite was 7.71% and 0.03% in the IC, and CTR steels, respectively. Moreover, the retained austenite in the IC steel was mostly distributed in bainite laths and at the grain boundaries. 

Considering that the accumulated deformation in the rough rolling process was apparently higher at 1/4-thickness of the IC steel than the CTR steel (Figure 8), the recrystallization of austenite grains was larger in volume fraction and resulted in refined austenite grains in the IC steel (Figure 5). Compared to the CTR steel, the larger austenite grain boundary area at 1/4-thickness of the IC steel could provide more nucleation sites for the ferrite during the phase transformation process. Thus, ferrite with low carbon density was larger in volume fraction at 1/4-thickness of the IC steel. Given that the carbon diffusion rate in ferrite (BCC, body-centered cubic) is higher than the fraction of retained austenite (FCC), the austenite with high carbon density gathers at the grain boundaries after the ferrite phase transformation. Hence, austenite with high carbon density was larger in volume fraction at 1/4-thickness of the IC steel than the CTR steel. Considering that the high content of alloy elements in the present steel inhibited the carbon diffusion, the austenite with high carbon density was quite stable. Therefore, the pearlite phase transformation was inhibited and M-A islands with a high proportion of retained austenite were easily formed.

Compared to the CTR steel, the large area austenite grain boundaries, in the IC steel, also enhanced the nucleation of bainite and, then, increased the phase transformation temperature, which increased the carbon diffusion rate in austenite and promoted the dislocation recovery in bainite. Therefore, carbon was able to diffuse faster into austenite from the interphase boundaries, between austenite and bainite, during the bainite phase transformation process, which inhibited the carbide precipitation at the interphase boundaries and enhanced the carbon concentration at the end of the bainite phase transformation. Consequently, dislocations and carbides in bainite were fewer and M-A islands, with high-density carbon, were more in IC steel than the CTR steel.

**Figure 14:**
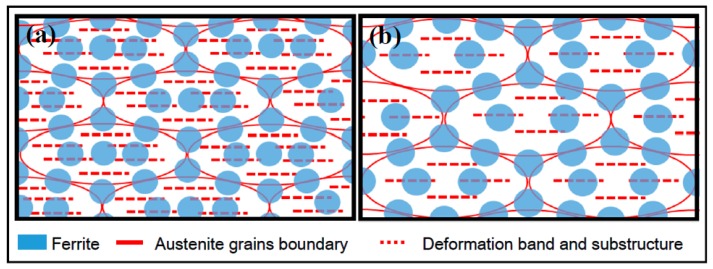
Schematic illustration of the ferrite phase transformation at (**a**) 1/4-thickness and (**b**) 1/2-thickness of IC steel.

Ferrite usually nucleates at the austenite grain boundaries, and nucleation sites of ferrite at the deformation bands inside the austenite grain increased with increasing cooling rate, while the acicular ferrite usually nucleates at the deformation bands inside the austenite grain. Thus, the ferrite nucleation inside the austenite grain was higher, due to the higher cooling rate and deformation at 1/4- than 1/2-thickness of the IC steel. Also, the area of austenite grain boundaries and deformation bands was large at 1/4-thickness of the IC steel. The schematic illustration of the ferrite phase transformations at 1/4- and 1/2-thickness of IC steel is presented in Figure 14. On the one hand, the ferrite grains nucleated inside the austenite grain could take up the nucleation sites of acicular ferrite. In addition, the large volume fraction of ferrite, formed in advance, could reduce the growing space of acicular ferrite and inhibit the acicular ferrite phase transformation, which results in the refinement of acicular ferrite lath at 1/4-thickness of the IC steel. On the other hand, at 1/2-thickness of the IC steel, the remaining austenite with low stability can be easily transformed into acicular ferrite, due to the reduced carbon concentration in the austenite, which resulted from the pearlite phase transformations, caused by the low cooling rate. Therefore, ferrite has shown a higher volume fraction and acicular ferrite has exhibited a lower volume fraction at 1/4- than at 1/2-thickness of the IC steel. 

### 4.2. Precipitation

The isothermal precipitation of Nb(C, N) in the Nb-containing steel has been studied [31], which can be explained by the given equation:

(10) $$t_{0.05}=3\times 10^{-6}{\left[Nb\right]}^{-1}\varepsilon^{-1}Z^{-0.5}\exp\frac{270000}{RT}\exp\frac{2.5\times 10^{10}}{T^{3}{\left(\ln K_{S}\right)}^{2}}$$

where $t_{0.05}$ refers to the time for 5% precipitation, [*Nb*] represents the volume fraction of Nb, ε denotes the strain, R represents the universal gas constant, *T* refers to the absolute isothermal temperature, *Z* corresponds to the Zener-Hollomon parameter and $K_{S}$ denotes the supersaturation ratio. *Z* and $K_{S}$ can be given by Equations (11) and (12) [32]:

(11) $$Z=\dot{\varepsilon }\exp\frac{400000}{RT_{\mathrm{def}}}$$

(12) $$K_{S}=\left[Nb\right]{\left[C+12N/14\right]}_{\mathrm{soln}}/10^{2.26-6770/T}$$

where $\dot{\varepsilon }$ refers to the strain rate and $T_{\mathrm{def}}$ represents the absolute deformation temperature. 

**Figure 15:**
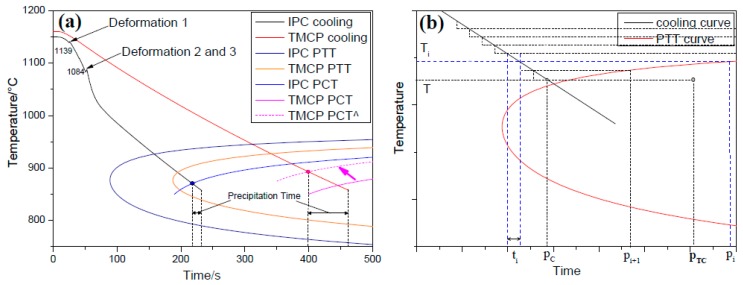
(**a**) Cooling curves, PTT (precipitation-time-temperature) curves and PCT (precipitation-cooling-temperature) curves at 1/4-thickness of both steel plates and (**b**) the schematic illustration of the accumulation law.

Combined with the simulation results, shown in Figure 8c and Table 3, Equation (10) can be expressed as a function of $t_{0.05}$ and *T*, which was used before the finish rolling process at 1/4-thickness of the two plates. The function of $t_{0.05}$ and *T* was drawn as the PTT (precipitation-time-temperature) curves and the results are shown in Figure 15a. 

During the cooling process, to investigate the precipitation behavior before the finish rolling, the PTT curves were transformed into PCT (precipitation-cooling-temperature) curves, by using the accumulation law [33], and the schematic diagram is presented in Figure 15b, where the cooling curve is divided into *n* equal isothermal parts. $t_{i}$ and $p_{i}$ correspond to the isothermal time and incubation time at temperature $T_{i}$. The accumulation law suggests that the accumulation of $t_{i}$/$p_{i}$ is the cooling time ($p_{C}$) proportion of the total incubation time ($p_{\mathrm{TC}}$) at temperature *T*. Thus, $p_{\mathrm{TC}}$ can be expressed by Equation (13):

(13) $$p_{\mathrm{TC}}=p_{C}/\overset{n}{\underset{i=1}{\sum }}\frac{t_{i}}{p_{i}}$$

The $p_{\mathrm{TC}}$-T curves (PCT) of the IC and CTR steels, at 1/4-thickness, are presented in Figure 15a.

As shown in Figure 8c, the deformation of IC steel plate during the rough rolling was significantly higher than the CTR steel. As a result, the inadequate recrystallization resulted in a higher density of dislocations and deformation bands in the austenite phase of the CTR steel, which reduced the incubation time and promoted the precipitation of NbC. As an indication, the PCT curve of CTR steel shifted to the upper left corner (dotted line in Figure 15a).

Therefore, the precipitation density of CTR steel is higher than the IC steel, due to the long precipitation time, as predicted by Figure 15a.

### 4.3. Tensile Strain Analysis

Owing to the presence of a large volume fraction of bainite and acicular ferrite, with a high density of mobile dislocations, the unpinning was not necessary for accumulated mobile dislocations [34,35], which is responsible for the continuous yielding, observed in tensile curves.

During plastic deformation of the IC steel, a large area of uniformly distributed ferrite could separate the bainite laths, with a high density of dislocations from each other, and disperse the stress concentration in the bainite matrix, which could delay the formation of early micro-cracks and lead to high crack initiation energy. Along with the dislocation accumulation in ferrite grains, stress in bainite matrix could hardly be released by the surrounded ferrite, which leads to the stress concentration at the interphase boundary of bainite and ferrite [36] and produces micro-cracks. The micro-cracks usually propagate along the high-angle grain boundaries [37], while macro-necking occurs, due to the interconnection of microcracks. Moreover, a number of micro-cracks have been simultaneously generated at the interphase boundary due to the higher large-angle grain boundary area of IC steel plate, as predicted in Figure 11a, which released the stress concentration and delayed the interconnection of micro-cracks. This contributes to the longer peak plateau length of the IC steel plate, as shown in Figure 9a.

It is mentioned above that there are three stages during the continuous yielding process of the studied steel [29,30]. The strain-hardening rate remained the lowest in the first stage, due to the low dislocation density in ferrite. During the second stage, piling up of the accumulated dislocations at the interphase boundary significantly improved the strain-hardening rate. In the third stage, the accumulated dislocations crossed the interphase boundary and triggered slip system in bainite, which released the stress concentration and, consequently, resulted in reduced strain-hardening rate. Ashby-Mileiko deformation theory demonstrates that the refined bainite lath and ferrite grain can increase the strain-hardening rate, resulting from the dislocation glide inhibition of high-angle grain boundaries. Owing to the larger high-angle grain boundary area of the IC steel, the strain-hardening rates in the three stages of the IC steel were higher than the CTR steel. At 1/4- and 1/2-thickness of the IC steel, the strain-hardening rate was lower at 1/2-thickness, in the first stage, due to the stress dispersion by the large area of ferrite (polygonal ferrite and acicular ferrite). In the second stage, the strain-hardening rate was higher at 1/4-thickness, due to the larger area of bainite. In the third stage, the strain-hardening rate was still higher at 1/4-thickness, resulting from larger high-angle grain boundaries area.

Owing to the large numbers of slip systems, the retained austenite with FCC structure absorbed and accommodated the high density of dislocations [38], which helped in reducing the dislocation pile-up. As a result, the formation of micro-cracks is delayed, which improved the crack initiation energy. Furthermore, the retained austenite with FCC structure has been transformed into martensite during severe deformation [39], which is indicated by the decrease in FCC volume fraction from 7.71% to 0.42% during tensile straining of the IC steel at 1/4-thickness. The phase transformation could absorb the energy of stress concentration during plastic deformation and the propagation energy of crack tips during necking. As a result, the necking process is delayed and overall ductility is enhanced [40]. All of these factors contributed to the ductility improvement of the IC steel at 1/4-thickness.

## 5. Conclusions

(1) Two cooling passes of 8 s and 12 s before the first and second rolling passes in the rough rolling process could dramatically improve the deformation penetration and refines the austenite grains inside the slab. The deformation amount and deformation rate in every deformation pass of rough rolling of IC steel were higher than the CTR steel.
(2) The number of austenite grains per unit area (mm^2^) in the IC steel (224) was larger than the CTR steel (140). The bainite content in the IC steel was significantly lower than the CTR steel. The volume fraction of ferrite and acicular ferrite in IC steel were 45% and 18%, at 1/4-thickness, and 35% and 50%, at 1/2-thickness, respectively. On the other hand, the sum of both phases was smaller than 25% in the CTR steel due to the coarsen austenite grains. Moreover, the high-angle grain boundary fraction in the IC steel was significantly higher than the CTR steel, which can be ascribed to the higher deformation amount in the IC steel.
(3) A large number of fine and shapeless pearlite has been observed in IC steel, whereas only large-sized carbides of the hexagonal structure have been found in CTR steel, which can be ascribed to the combination of ferrite volume fraction and cooling rate. Compared to the CTR steel, the density of precipitates in the IC steel was lower due to the lower density of dislocations and deformation bands in the austenite phase and the shorter time before the finish rolling. In addition, two kinds of Nb containing precipitates, such as (Ti, Nb)(C, N) and (Nb, Ti)C, have been observed in the studied steels.
(4) Total ductility and uniform elongation of the IC steel were higher than the CTR steel, while the crack initiation energy and crack propagation energy during tensile straining of the IC steel were higher than the CTR steel at 1/4-thickness, which can be ascribed to the high volume fraction of refined ferrite with low-density dislocation. In addition, the volume fraction of retained FCC austenite phase has been reduced from 7.71% to 0.42% near the tensile fracture region in IC steel at 1/4-thickness, which can contribute to the ductility improvement.
(5) During the continuous yielding process, three strain hardening stages at 1/4- and 1/2-thickness of IC and CTR steel plates have been observed. The strain-hardening rate was maximum during the second stage and minimum during the first stage. In the third stage, the strain of synergistic plastic deformation in the IC steel was higher than the CTR steel. Meanwhile, the synergistic plastic deformation of the IC steel occurred at lower stress, after yielding of the plate, than the CTR steel, which can be ascribed to the presence of lesser micro-cracks in the IC steel. Thus, the lower density of micro-cracks delayed fracture of IC steel plate.

## Figures and Tables

**Table 1:** The main parameters used in the simulations.

Parameter	Value
Roll diameter (mm)	750
Rolling speed (rad/sec)	1.33
Initial temperature of roller (°C)	20
Initial temperature of slab (°C)	1150
Young’s modulus (GPa)	210
Poisson’s ratio	0.35
Deformation resistance (MPa)	Equation (1)
Shear friction coefficient	Equation (2)
Heat convection coefficient in air cooling process (m^2^·°C)	200
Heat convection coefficient in water cooling process (m^2^·°C)	5000

**Table 2:** Statistics of size and number density of austenite grains.

Process	Average Grain Size along Thickness Direction (μm)	Grain Quantity Unit Volume (mm^2^)	Average Length-Width Ratio
CTR	70	140	2.35
IC	37	224	3.34

**Table 3:** Deformation temperature and deformation rate of the rolled plates at 1/4-thickness.

Process	Deformation Temperature/°C			Deformation Rate/s		
	Td1	Td2	Td3	Rate1	Rate2	Rate3
IC	1139	1084	1084	1.8	2.2	2
CTR	1150	1150	1150	1.63	1.93	1.87

**Table 4:** Mechanical parameters and energy distribution during uniaxial tensile testing of IC and CTR plates at 1/4-thickness.

Process	$\varepsilon_{TY}$	$\varepsilon_{TP}$	$\varepsilon_{TC}$	$\sigma_{TY}$	$\sigma_{TP}$	$\sigma_{TC3}$	$\sigma_{TC1}$	*n*	*K*	$E_{e}$	$E_{p}$	$E_{c}$
**IC**	0.004	0.094	1.59	453	655	324	219	0.12	866	0.91	52.6	655
**CTR**	0.004	0.072	1.557	456	641	312	212	0.1	827	0.91	39.8	633

**Table 5:** Statistics of the critical parameters, derived from Figure 10.

Process	*m*_1_	*m*_2_	*m*_3_	Stress of A (MPa)	Stress of B (MPa)	$\sigma_{B}$ − $\sigma_{A}$ (MPa)	$\varepsilon_{C}$ − $\varepsilon_{B}$
CTR 1/4	14	9.1	10.7	455	555	100	0.052
IC 1/4	11.7	5.6	7	450	528	78	0.09
CTR 1/2	14.1	7.2	11.5	462	561	99	0.063
IC 1/2	12.6	6.9	9.9	466	550	84	0.065
